# Non cell-autonomous role of DCC in the guidance of the corticospinal tract at the midline

**DOI:** 10.1038/s41598-017-00514-z

**Published:** 2017-03-24

**Authors:** Quentin Welniarz, Marie-Pierre Morel, Oriane Pourchet, Cécile Gallea, Jean-Charles Lamy, Massimo Cincotta, Mohamed Doulazmi, Morgane Belle, Aurélie Méneret, Oriane Trouillard, Marta Ruiz, Vanessa Brochard, Sabine Meunier, Alain Trembleau, Marie Vidailhet, Alain Chédotal, Isabelle Dusart, Emmanuel Roze

**Affiliations:** 1 Sorbonne Universités, UPMC Univ Paris 06, INSERM U 1127, CNRS UMR 7225, Institut du Cerveau et de la Moelle épinière, F-75013 Paris, France; 2 Sorbonne Universités, UPMC Univ Paris 06, INSERM, CNRS, Institut de Biologie Paris Seine, Neuroscience Paris Seine, F-75005 Paris, France; 3 Unità Operativa di Neurologia-Firenze, Azienda USL Toscana Centro, Ospedale San Giovanni di Dio, 50143 Firenze, Italy; 4 Sorbonne Universités, UPMC Univ Paris 06, INSERM, CNRS, Institut de Biologie Paris Seine, Adaptation Biologique et vieillissement, F-75005 Paris, France; 5 Sorbonne Universités, UPMC Univ Paris 06, INSERM, CNRS, Institut de la Vision, F-75012 Paris, France; 6 Centre d’Investigation Clinique 14-22, INSERM/AP-HP, Paris, France; 7 Département de Neurologie, AP-HP, Hôpital Pitié Salpêtrière, Paris, France

## Abstract

DCC, a NETRIN-1 receptor, is considered as a cell-autonomous regulator for midline guidance of many commissural populations in the central nervous system. The corticospinal tract (CST), the principal motor pathway for voluntary movements, crosses the anatomic midline at the pyramidal decussation. CST fails to cross the midline in *Kanga* mice expressing a truncated DCC protein. Humans with heterozygous *DCC* mutations have congenital mirror movements (CMM). As CMM has been associated, in some cases, with malformations of the pyramidal decussation, DCC might also be involved in this process in human. Here, we investigated the role of DCC in CST midline crossing both in human and mice. First, we demonstrate by multimodal approaches, that patients with CMM due to *DCC* mutations have an increased proportion of ipsilateral CST projections. Second, we show that in contrast to *Kanga* mice, the anatomy of the CST is not altered in mice with a deletion of DCC in the CST. Altogether, these results indicate that DCC controls CST midline crossing in both humans and mice, and that this process is non cell-autonomous in mice. Our data unravel a new level of complexity in the role of DCC in CST guidance at the midline.

## Introduction

The corticospinal tract (CST) is the principal motor pathway for voluntary movements^1–3^. Most CST axons cross the midline at the junction between the brainstem and spinal cord, thereby forming the pyramidal decussation. To cross the midline, central nervous system (CNS) axons are guided by molecular cues whose expression, together with that of their receptors, is tightly controlled in time and space during development^4, 5^. DCC (Deleted in Colorectal Cancer) is a receptor that mediates the chemoattractive activity of NETRIN-1, thereby modulating the crossing of CNS commissural axons^6^. In *Dcc* ^−/−^ knockout mice, midline crossing by commissural axons is altered at the level of the corpus callosum (CC), anterior commissure, hippocampal commissure^7–9^, habenulo-interpeduncular system^10^, inferior olive^11^, and spinal cord^7, 12^. DCC is considered as a cell-autonomous regulator for midline crossing, as many commissural neurons that express DCC fail to cross the midline in *Dcc* mutants^7–9, 12^.

The role of DCC in the development of the CST has not been investigated in *Dcc* ^−/−^ knockout mice. Indeed, they die within 24 hours after birth, when the CST crosses the midline and enter the spinal cord. *Dcc* ^*kanga*^ mice carry a spontaneous and viable *Dcc* mutation that removes the exon encoding the P3 intracellular domain^8^. The study of *Dcc* ^*kanga*^ mice provided evidence supporting a role of DCC in the development of the mouse CST. These mice are characterized by a striking “kangaroo-like” hopping gait, and replicate most of the commissural defects observed in *Dcc* ^−/−^ mutants. At the level of the pyramidal decussation, the CST of *Kanga* mutants does not cross the midline but forms two bundles that remain in the ventral ipsilateral spinal cord^8^. However, DCC has not been detected in brainstem CST axons during normal development^8, 13^, raising the possibility that DCC might influence CST midline crossing in a non cell-autonomous manner.

In human, heterozygous mutations in *DCC* have been identified in families with autosomal-dominant congenital mirror movements (CMM)^14–16^. Mirror movements (MM) are involuntary symmetrical movements of one hand that mirror voluntary movements of the other hand. CMM is associated with malformations of the pyramidal decussation, at least in some cases^17–19^. Two CMM patients with initially unknown genetic status were eventually found to carry a *DCC* mutation, years after publication of their neurophysiological data. In these two patients, unilateral transcranial magnetic stimulation (TMS) of the primary motor cortex elicited bilateral motor responses, suggesting the existence of bilateral CST projections to the spinal cord^20–22^. However, further neurophysiological and neuroimaging data are needed to validate these results and to clarify to what extent the pyramidal decussation is morphologically and functionally abnormal in *DCC*-CMM patients.

The aim of the present paper was to study the role of DCC in CST midline crossing in both human and mice: we checked whether the role of DCC in the development of the pyramidal decussation is conserved in human and whether this process is cell-autonomous in mouse. First, we used an optimized multimodal approach to characterize in details the abnormalities of the CST in a group of six *DCC*-CMM patients. Second, we studied the motor consequences of *Dcc* mutations in mice. Last, for the first time, we used conditional *Dcc* mouse mutants to unravel the role of DCC in axon guidance at the midline. We analyzed the anatomy of the CST in various *Dcc* deficient mouse mutants, including a line with a conditional deletion of DCC in the neocortex (and thus in the CST).

## Results

### Abnormal ipsilateral corticospinal projections in *DCC*-CMM patients

**Figure 1:**
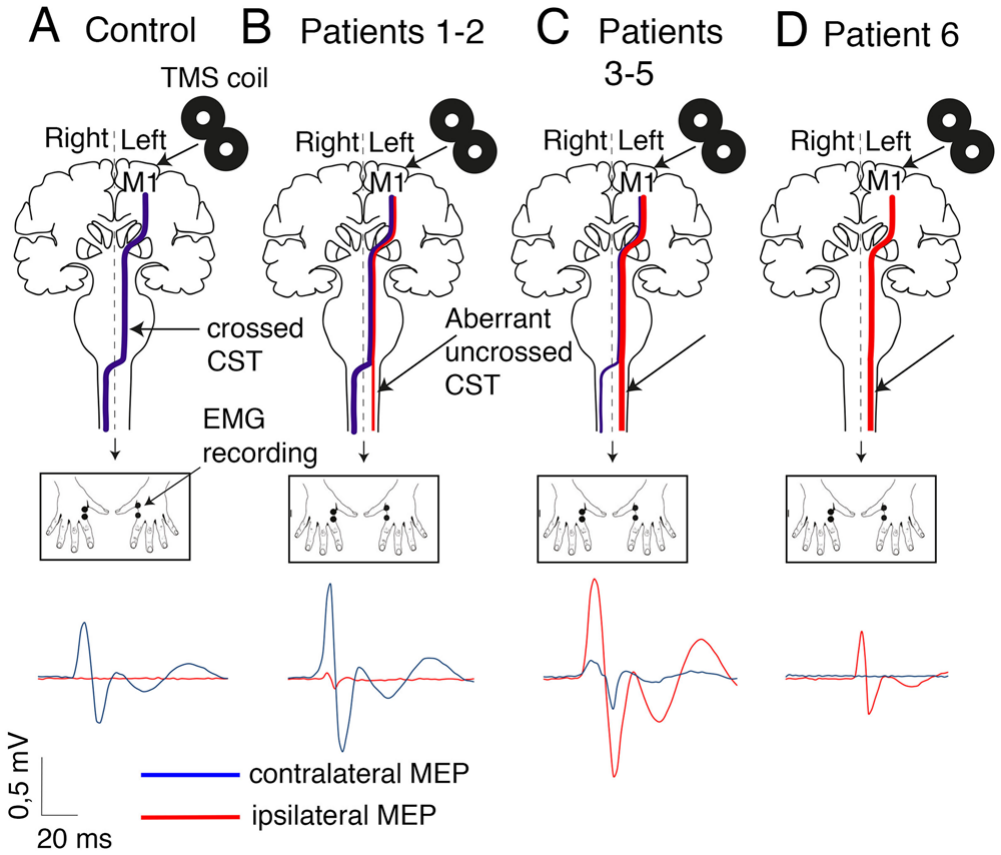
In healthy volunteers, unilateral stimulation of the hand area of the dominant primary motor cortex elicited only contralateral MEPs (**A**, blue line, right hand), whereas ipsilateral MEPs were observed in the *DCC* patients (**B–D**, red line, left hand). Depending on the patient, the ipsilateral MEPs were smaller (**B**) or larger (**C**) than the contralateral MEPs. In patient #6, stimulation of the dominant hemisphere elicited only ipsilateral MEPs (**D**).

To determine whether DCC is involved in the formation of the pyramidal decussation in humans, we first studied six patients with typical congenital mirror movements due to *DCC* mutations^14, 16^. In these patients, intentional movements of one hand are accompanied by involuntary mirror movements of the other hand (Supplementary Movie 1). The patients had no additional clinical manifestations. We used single-pulse TMS to investigate how neural signals propagate along the CST (Table 1 and Fig. 1). In the healthy controls, stimulation of the cortical representation of hand muscles at rest elicited contralateral responses only (Fig. 1A). In contrast, in the six *DCC*-CMM patients, unilateral stimulation of the primary motor cortex at rest elicited ipsilateral responses, which were absent in all six controls. This suggested the existence of fast-conducting corticospinal projections from the hand area of the dominant primary motor cortex to motoneurons on the ipsilateral side of the spinal cord in the patients^17, 18^. Ipsilateral MEPs were observed in 100% of the pulses in five *DCC*-CMM patients, and in 65% of the pulses in the remaining patient (Table 1).

**Table 1:** Frequency, amplitude and latency of the ipsilateral MEPs in *DCC*-CMM patients

Subject	Family Mutations	Gender/Age	MM WT score	Frequency of ipsilateral MEPs	Relative amplitude (MEPipsi/MEPcontra)	Latency of contralateral MEPs (ms)	Latency of ipsilateral MEPs (ms)
Patient 1	Family 1: Exon 4 c.823 C > T /p.Arg275*	M/41	2	65%	4%	22,8	23,6
Patient 2	Family 2: Exon 26 c.3835_3836del/p.Leu1279Profs*	F/51	3	100%	47%	21,9	22,5
Patient 3	Family 3: Exon 4 c.823 C > T /p.Arg275*	M/42	2	100%	392%	25,1	24,9
Patient 4	Family 2: Exon 26 c.3835_3836del/p.Leu1279Profs*	M/49	3	100%	322%	23,0	22,7
Patient 5	Family 4: del DCCex4and5	F/79	3	100%	409%	22,5	22,1
Patient 6	Family 1: Exon 4 c.823 C > T /p.Arg275*	F/44	2	100%	ipsilateral MEPs only		20,7

The frequency of ipsilateral MEPs represents the percentage of trials in which unilateral stimulation of the dominant hemisphere elicited ipsilateral muscular responses. MEP: motor evoked potentials; MM: mirror movements; WT: Woods and Teuber.

**Figure 2:**
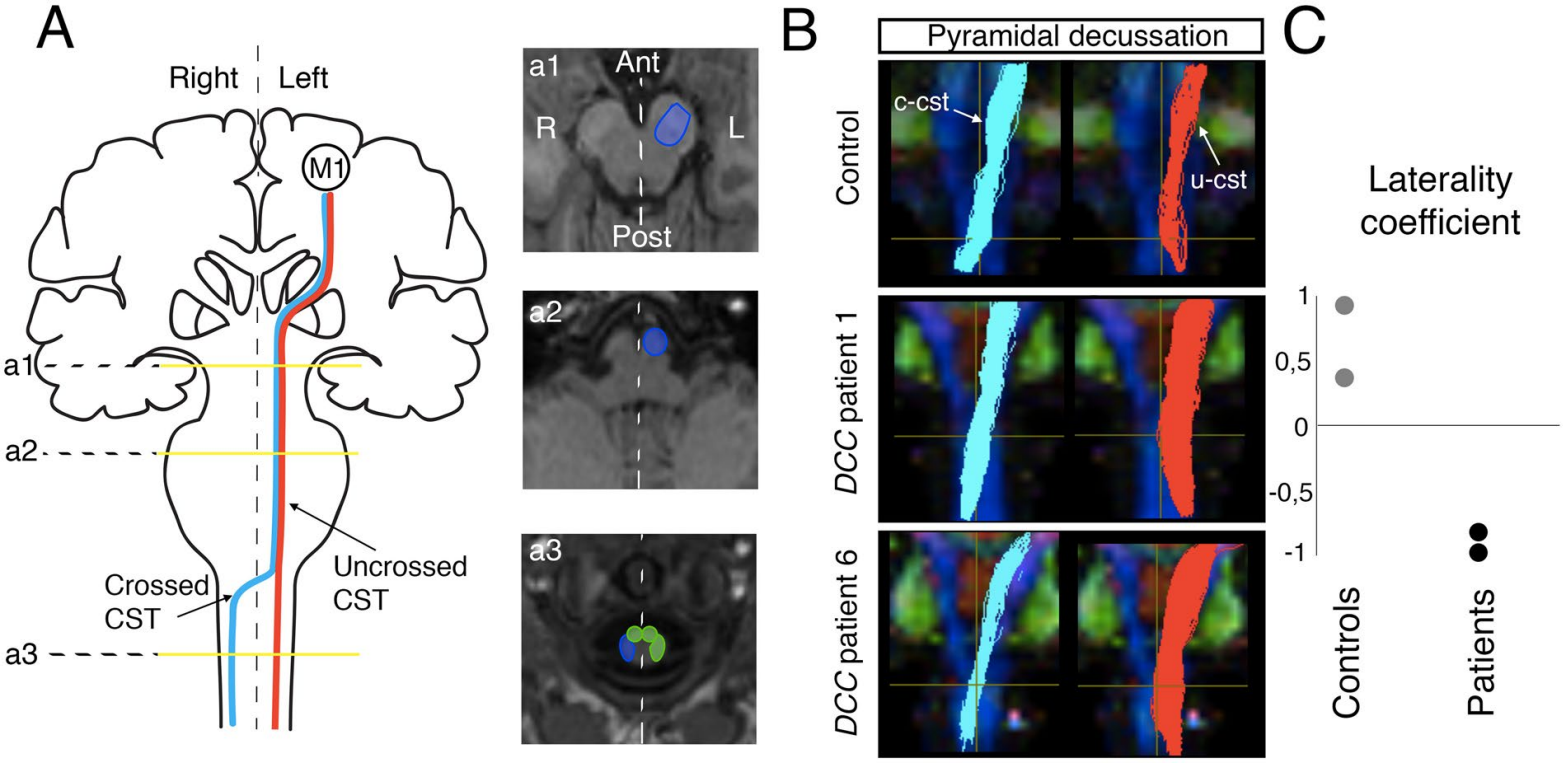
Tractography of the CST in *DCC*-CMM patients. (**A**) Left: color coding of the crossed (blue) and uncrossed (red) corticospinal tracts; Right: regions of interest (both in the diagram and superimposed on an axial slice of an anatomical image of a subject) used to reconstruct the fiber tracts (blue area) at the base of the pons (a1), the anterior pyramid in the upper medulla (a2), and the crossed lateral funiculus of the upper cervical cord (a3). The crossed CST from the left M1 to the right upper cervical cord was reconstructed after excluding fibers reaching the right medial and left lateral and medial funiculi (green area). (**B**) Tractography of the corticospinal tract superimposed on the individual fractional anisotropy color map of a control subject and two *DCC*-CMM patients. Individual coronal views at the level of the decussation are presented. Light blue tracts represent the crossed CST, and red tracts the uncrossed CST. (**C**) The corticospinal tract laterality coefficient is expressed as (NF Crossed − NF Uncrossed)/(NF Crossed + NF Uncrossed), where NF is the number of fibers. The coefficient was positive for the two controls (indicating more fibers in the crossed corticospinal tract) and negative for the two *DCC*-CMM patients (indicating more fibers in the uncrossed corticospinal tract).

The relative amplitude of the contralateral and ipsilateral MEPs was variable in the *DCC*-CMM patients. The amplitude of the ipsilateral MEPs was smaller than the normal contralateral MEPs in two of the six patients (Fig. 1B, Table 1) and larger in three of the six patients (Fig. 1C, Table 1), whereas only ipsilateral MEPs were observed in one patient (Fig. 1D, Table 1). This variability in the amplitude of ipsilateral and contralateral MEPs could reflect differences in the relative number of ipsilateral and contralateral CST projections to the spinal cord. In patients #1–5, who had bilateral MEPs, the difference in latency between ipsilateral and contralateral MEPs was less than 1 millisecond (Table 1), which is consistent with the presence of direct corticospinal projections from the dominant hemisphere to the ipsilateral spinal cord. We then used diffusion tensor imaging (DTI) to investigate the CST projections in two patients and two controls (Fig. 2). The results suggested that the controls had more crossed CST fibers than uncrossed CST fibers, whereas the patients had more uncrossed CST fibers than crossed CST fibers (Fig. 2B,C). Together, these findings support the involvement of DCC in the development of the pyramidal decussation in humans, as previously observed in mice.

### The study of *Dcc*^*kanga*/*−*^ mice highlights the role of DCC in the generation of asymmetric movements

**Figure 3:**
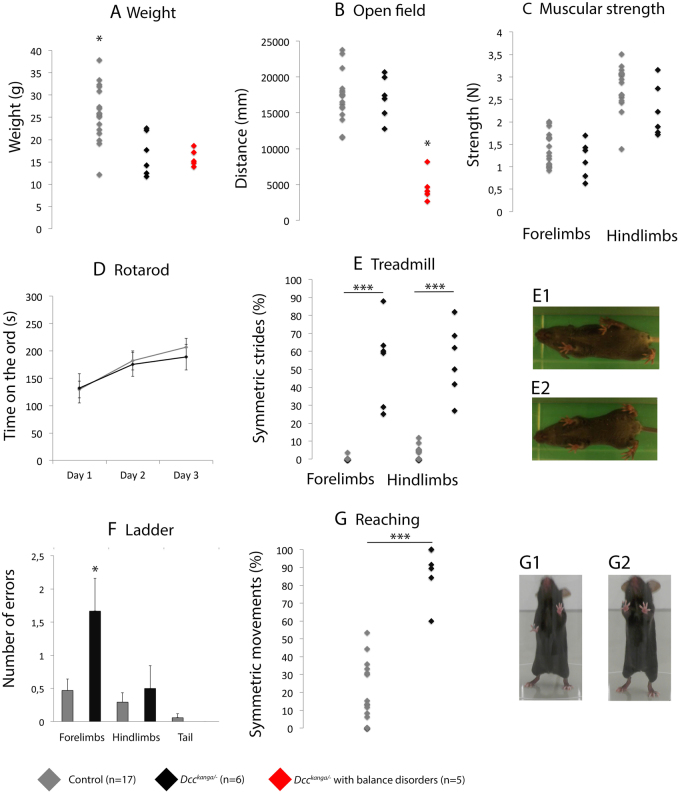
DCC is required for asymmetric movements. We used behavioral tests to investigate the motor phenotype of *Dcc* ^*kanga*/*−*^ mutant mice. *Dcc* ^*kanga*/*−*^ mice (n = 11; black and red) were compared to *Dcc* ^+/+^ mice or *Dcc* ^*kanga*/+^ mice (that behave like wildtype mice, n = 17; gray). Five of the 11 *Dcc* ^*kanga*/*−*^ mice displayed marked balance disorders (red): they were unable to stand on their limbs and thus moved very little in the open-field test (ANOVA F_(2,25)_ = 33.18, p < 0.001, followed by the Bonferroni *post hoc* test; **B**). Because they were unable to perform most of the motor tests, they were excluded from further analysis. *Dcc* ^*kanga*/*−*^ mice were lighter than their littermate controls (ANOVA F_(2,20)_ = 6.27, p = 0.008, followed by the Bonferroni *post hoc* test; **A**) and were accordingly weaker in the muscle strength test (Student’s test, p_forelimbs_ = 0.228; p_hindlimbs_ = 0.042; **C**). *Dcc* ^*kanga*/*−*^ mice were indistinguishable from controls in the Rotarod test (repeated-measures ANOVA with two factors. F_(1,21)_ = 0.71, p = 0.793, followed by the Bonferroni *post hoc* test; **D**). On the treadmill, *Dcc* ^*kanga*/*−*^ mice displayed a striking hopping gait, frequently moving both their forelimbs and their hindlimbs simultaneously (Mann-Whitney test, p_forelimbs_ < 0.0001; p_hindlimbs_ < 0.0001; **E**, E2). In contrast, control mice made alternating movements (E, E1) of their forelimbs and hindlimbs. In the ladder test, *Dcc* ^*kanga*/*−*^ mice made more forelimb errors than the controls (Freeman-Halton extension of Fisher’s exact test, p = 0.038; **F**). When placed in a new walled environment, mice have a tendency to establish contacts on the walls with their forelimbs in an asymmetric (G1, control mice) or symmetric (G2, *Dcc* ^*kanga*/*−*^ mice) manner. In the reaching test, *Dcc* ^*kanga*/*−*^ mice made more symmetric forelimb movements than the controls (Student’s test, p < 0.0001; **G**).

*Dcc* ^*kanga*^ is a spontaneous mutation that removes the exon encoding the P3 intracellular domain of DCC, resulting in abnormal pyramidal decussation. *Dcc* ^*kanga*/*kanga*^ mice present a hopping gait and some of them have ataxia^8^. The human MM and the rodent hopping gait are two conditions characterized by the inability to produce asymmetric movements, but they are not equivalent. Indeed, it was recently shown that left-right alternation during locomotion relies on spinal commissural circuits rather than on proper CST wiring^23, 24^. This suggests that the hopping gait is not related to the abnormal anatomy of the CST in *Dcc* ^*kanga*/*kanga*^ mice. In order to further characterize the motor phenotype of *Dcc* ^*kanga*^ mice, we used a battery of motor tests, and in particular the exploratory reaching test recently used to evaluate the role of the CST in motor lateralization^23–25^. As we were not able to generate *Dcc* ^*kanga*/*kanga*^ mice, *Dcc* ^*kanga*/+^ mice were crossed with *Dcc* ^+/−^ mice to obtain *Dcc* ^*kanga*/*−*^ mice, in which one allele bears a Kanga mutation and the other is deleted. We therefore compared the performance of *Dcc* ^*kanga*/*−*^ mice with control mice (wildtype or *Dcc* ^*kanga*/+^) with various motor tests. Five of eleven *Dcc* ^*kanga*/*−*^ mice had major balance disorders: they were unable to stand on their limbs and therefore moved very little during the open-field test (ANOVA F_(2,25)_ = 33.18, p < 0.001, followed by the Bonferroni *post hoc* test; Fig. 3B). Because they were unable to perform most of the motor tests, they were excluded from further analysis. *Dcc* ^*kanga*/*−*^ mice were lighter than their littermate controls (ANOVA F_(2,20)  _ = 6.27, p = 0.008, followed by the Bonferroni *post hoc* test, Fig. 3A), and therefore did not perform as well as the controls in the muscle strength test (Student’s test, p_forelimbs_ = 0.228; p_hindlimbs_ = 0.042; Fig. 3C). *Dcc* ^*kanga*/*−*^ mice were undistinguishable from controls in the Rotarod test (repeated-measures ANOVA with two factors, F_(1,21)_ = 0.71, p = 0.793, followed by the Bonferroni *post hoc* test; Fig. 3D). On the treadmill test, *Dcc* ^*kanga*/*−*^ mice displayed a hopping gait, frequently moving both their forelimbs and hindlimbs simultaneously (Mann-Whitney test, p_forelimbs_ < 0.0001; p_hindlimbs_ < 0.0001; Fig. 3E,E2; Supplementary Movie 2). In contrast, the control mice made alternating movements with their forelimbs and hindlimbs (Fig. 3E,E1; Supplementary Movie 3). In the ladder test, which evaluates the precision of limb positioning, *Dcc* ^*kanga*/*−*^ mice made more forelimb errors than controls (Freeman-Halton extension of Fisher’s exact test, p = 0.038; Fig. 3F). The exploratory reaching test evaluates the lateralization of voluntary forelimb movements. When placed in a new walled environment, mice have a tendency to establish contacts on the walls with their forelimbs in an asymmetric (Fig. 3G,G1; Supplementary Movie 4) or symmetric (Fig. 3G,G2; Supplementary Movie 5) manner. In the reaching test, *Dcc* ^*kanga*/*−*^ mice made more symmetric movements of the forelimbs than the controls (Student’s test, p < 0.0001; Fig. 3G). The motor phenotype of *Dcc* ^*kanga*/*−*^ mice underlines the importance of DCC in the generation of alternating movements during locomotion, and of voluntary asymmetric forelimb movements. Altogether, our results demonstrate the link between DCC deficiency and the ability to produce asymmetric voluntary movements in both human and mice.

### DCC expression in CST axons is not required for midline crossing

**Figure 4:**
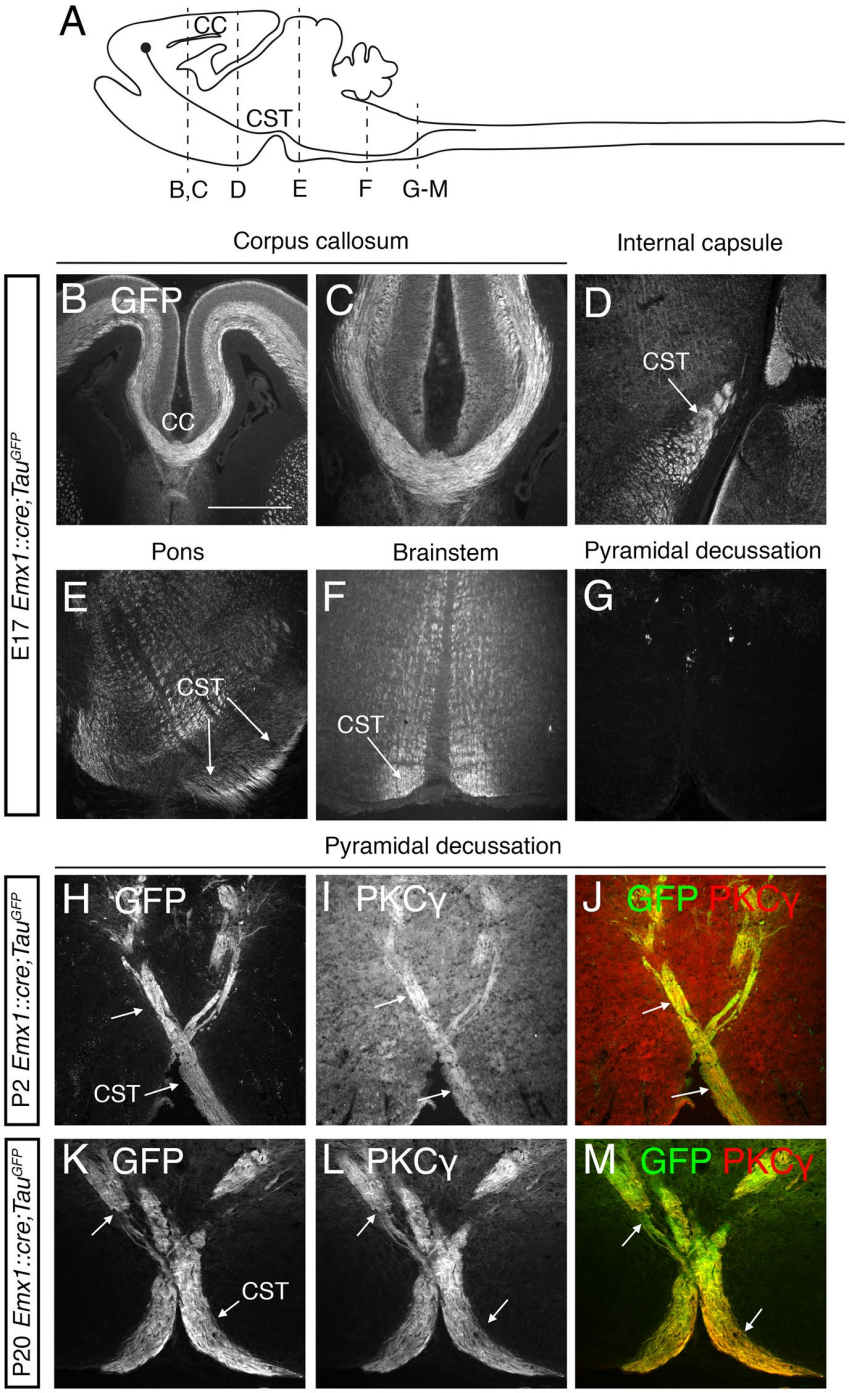
EMX1 is expressed in Corpus callosum and CST neurons before they cross the midline. (**A**) Schematic representation of a sagittal section through the CNS of a P2 mouse indicating the trajectory of the CST and the level of the coronal sections presented in this figure. Coronal sections of E17 (n = 3; B–G), P2 (n = 3; H–J) and P20 (n = 3; **K–M**) *Emx1*::*cre*;*Tau* ^*GFP*^ mice stained with anti-GFP (**B–M**) and anti-PKCγ, a marker of the CST (**H–M**). At E17, GFP was expressed in the corpus callosum axons at the midline (**B, C**). GFP staining was detected along the entire trajectory of the CST axons: in the internal capsule (**D**); at the level of the pons, where the CST adopts a ventral position (**E**); and in the brainstem, in a ventral position (**F**). At this age, the CST has not yet reached the pyramidal decussation (**G**). At P2 and P20, GFP staining co-located with PKCγ staining at the level of the pyramidal decussation (**H–M**). White arrows indicate CST axons. The scale bar represents 336 μm in B and 168 μm in (C-M).

**Figure 5:**
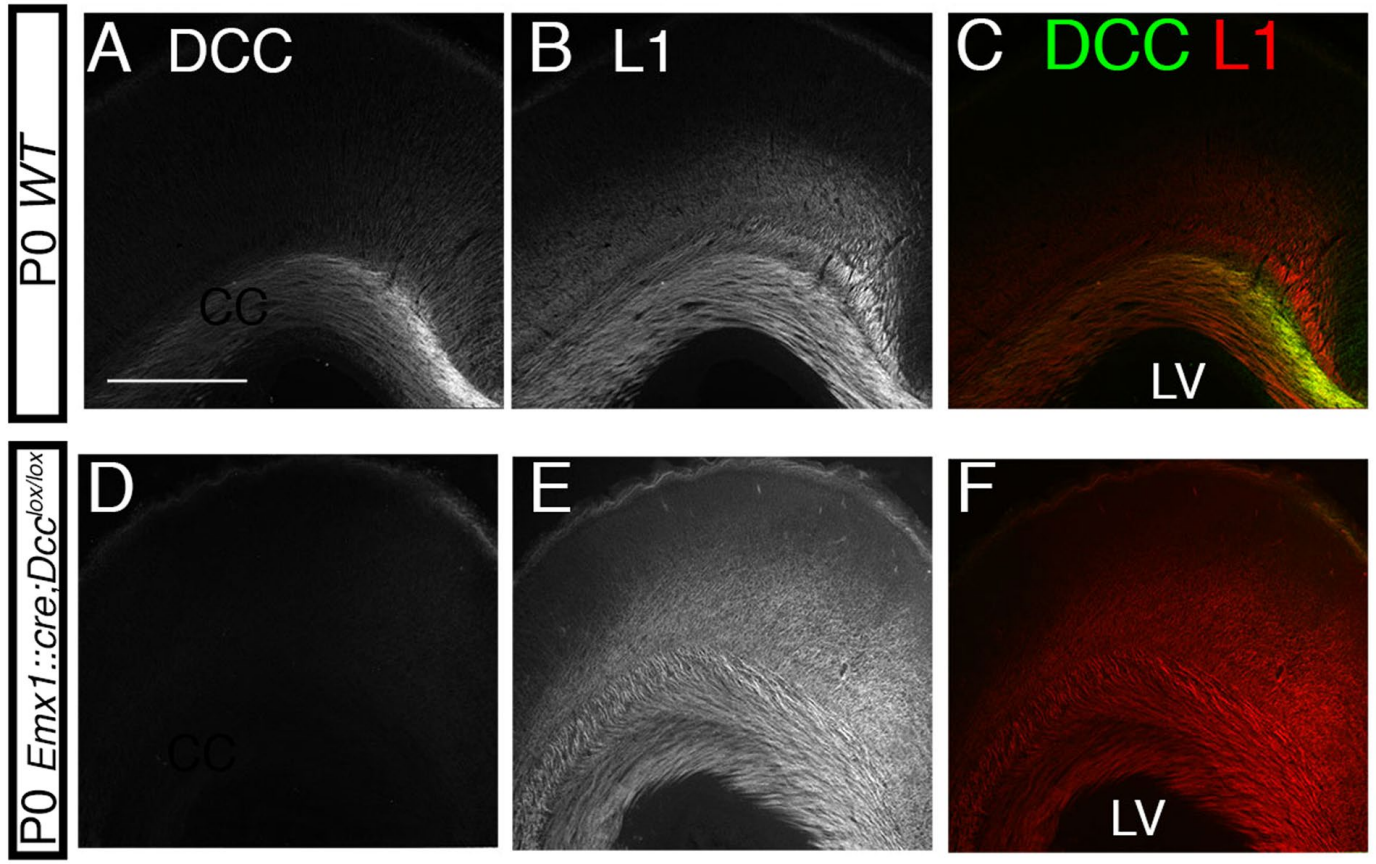
DCC is expressed in the neocortex and CC of wild type mice but not in *Emx1*::*cre*;*Dcc* ^*lox*/*lox*^ mice. Coronal sections of P0 wild type mice (n = 3; **A–C**) and of P0 *Emx1*::*cre*;*Dcc* ^*lox*/*lox*^ mice (n = 3; **D–F**) stained with anti-DCC and anti-L1. At P0, DCC was detected in the neocortex and CC of wild type mice (**A–C**). However, it was not detected in the neocortex of *Emx1*::*cre*;*Dcc* ^*lox*/*lox*^ mice (**D–F**). The scale bar represents 168 μm in A–F. LV: lateral ventricle.

**Figure 6:**
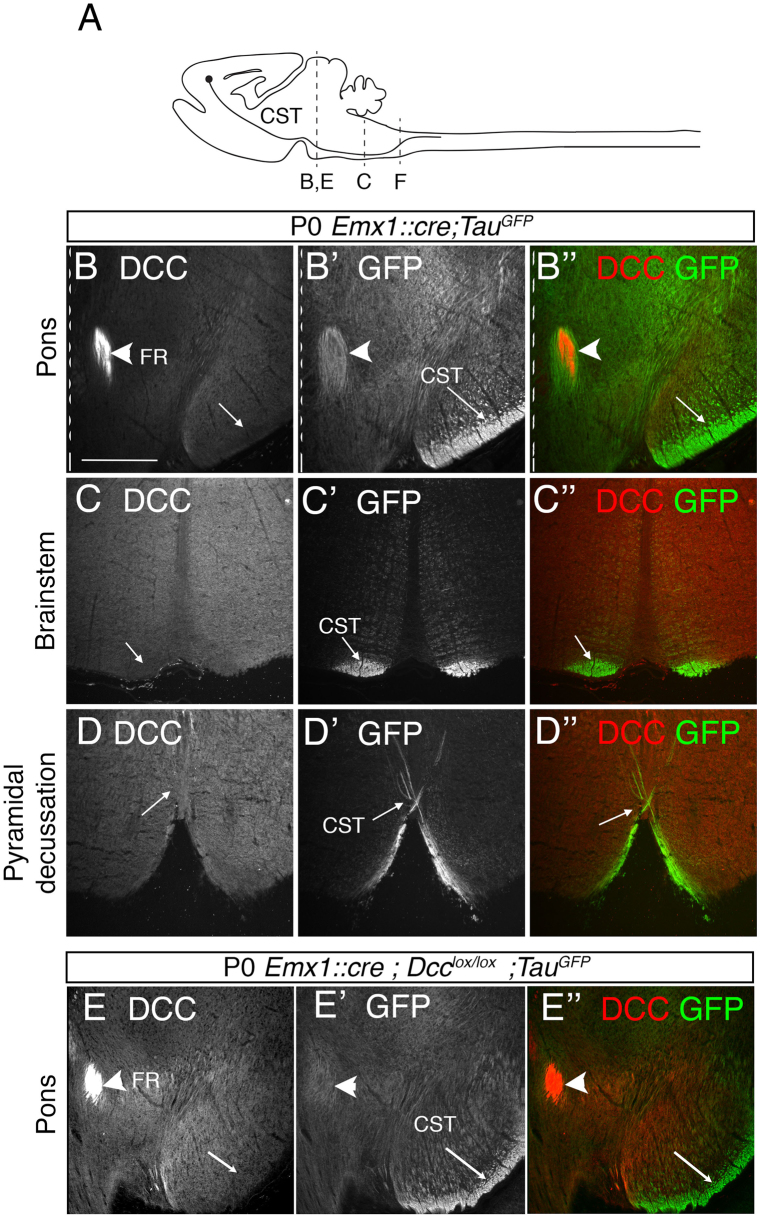
DCC is not detected in CST axons when they cross the midline. (**A**) Schematic representation of a P0 mouse sagittal section indicating the trajectory of the CST and the level of the coronal sections presented in this figure. Coronal sections of P0 *Emx1*::*cre*;*Tau* ^*GFP*^ mice (n = 3, **B**, **C**, **D**) and of P0 *Emx1*::*cre*;*Dcc* ^*lox*/*lox*^;*Tau* ^*GFP*^ mice (n = 3, **E**) stained with anti-DCC (**B–E**), anti-GFP (B’–E’), and both labels (B”–E”). GFP staining was used as a marker of corticospinal tract axons. DCC was not detected at the CST in the pons (**B,E**), brainstem (**D**) or pyramidal decussation (**F**). DCC was expressed in the fasciculus retroflexus (FR, **B**,**E**), used as a positive control. Dashed lines indicate the midline. The scale bar represents 184 μm.

We then investigated whether the CST midline crossing defects of *Dcc* ^*kanga*^ mice may result from a cell autonomous effect of DCC. This point remains an open question since DCC has not been detected in brainstem CST axons during normal development^8, 13^. To address this issue, we selectively abolished *Dcc* expression in cortical neurons by using *Dcc* conditional knockout mice^26^ and the *Emx1*::*cre* mouse line^27^. Before comparing the CST trajectory in the different mouse lines, we performed various control experiments. We first verified that EMX1 was expressed in corpus callosum (CC) and CST neurons before they crossed the midline (at E17 and P0, respectively)^28–30^. *Emx1*::*Cre* mice were first crossed with Tau-lox-Stop-lox-mGFP-IRES-nls-lacZ mice (*Tau* ^*mGFP*^), which express a membrane-tethered GFP in axons following Cre-mediated recombination^31^. In E17 *Emx1*::*cre*;*Tau* ^*GFP*^ mice, GFP expression was detected within corpus callosum axons at the midline (Fig. 4B,C) and at the described location of CST axons (Fig. 4D–F). At this stage, the CST has not yet reached the pyramidal decussation (Fig. 4G). After P2, CST axons can be identified by protein kinase C gamma (PKCγ) immunohistochemistry^32, 33^. We confirmed that CST axons were GFP-positive by dual immunostaining for GFP and PKCγ at P2 (Fig. 4H–J) and P20 (Fig. 4K–M). Then, we demonstrated that DCC is conditionally deleted in *Emx1*::*cre*;*Dcc* ^*lox*/*lox*^ cortical neurons (Fig. 5). Accordingly, we show that both *Dcc* ^*kanga*/*−*^ (n = 3/3) and *Emx1*::*cre*;*Dcc* ^*lox*/*lox*^ (n = 3/3) adult mice lacked the CC (Figure Supp 1)^7, 8^. Altogether these data demonstrate that EMX1 is expressed in CST neurons before their axons cross the midline and that DCC is lacking in the neocortex (and thus in the CST) of *Emx1*::*cre*;*Dcc* ^*lox*/*lox*^ mice. We then analyzed the expression of DCC along the CST at P0 (i.e. when pioneer CST axons reach the pyramidal decussation in mice^29, 34^) on *Emx1*::*cre*;*Tau* ^*GFP*^ mice. DCC was not detected in the CST of *Emx1*::*cre*;*TauGFP* either at the level of the pons (Fig. 6B), in the brainstem (Fig. 6C) or at the pyramidal decussation (Fig. 6D), in keeping with previous observations^8, 13^. Last we showed that, in both *Emx1*::*cre*;*Tau* ^*GFP*^ and *Emx1*::*cre*;*DCC* ^*lox*/*lox*^;*Tau* ^*GFP*^ mouse lines, DCC was detected in the fasciculus retroflexus (FR, Fig. 6B,C), demonstrating our ability to detect DCC and the fact that the expression of DCC is still present outside the neocortex in *Emx1*::*cre*;*Dcc* ^*lox*/*lox*^ mice.

**Figure 7:**
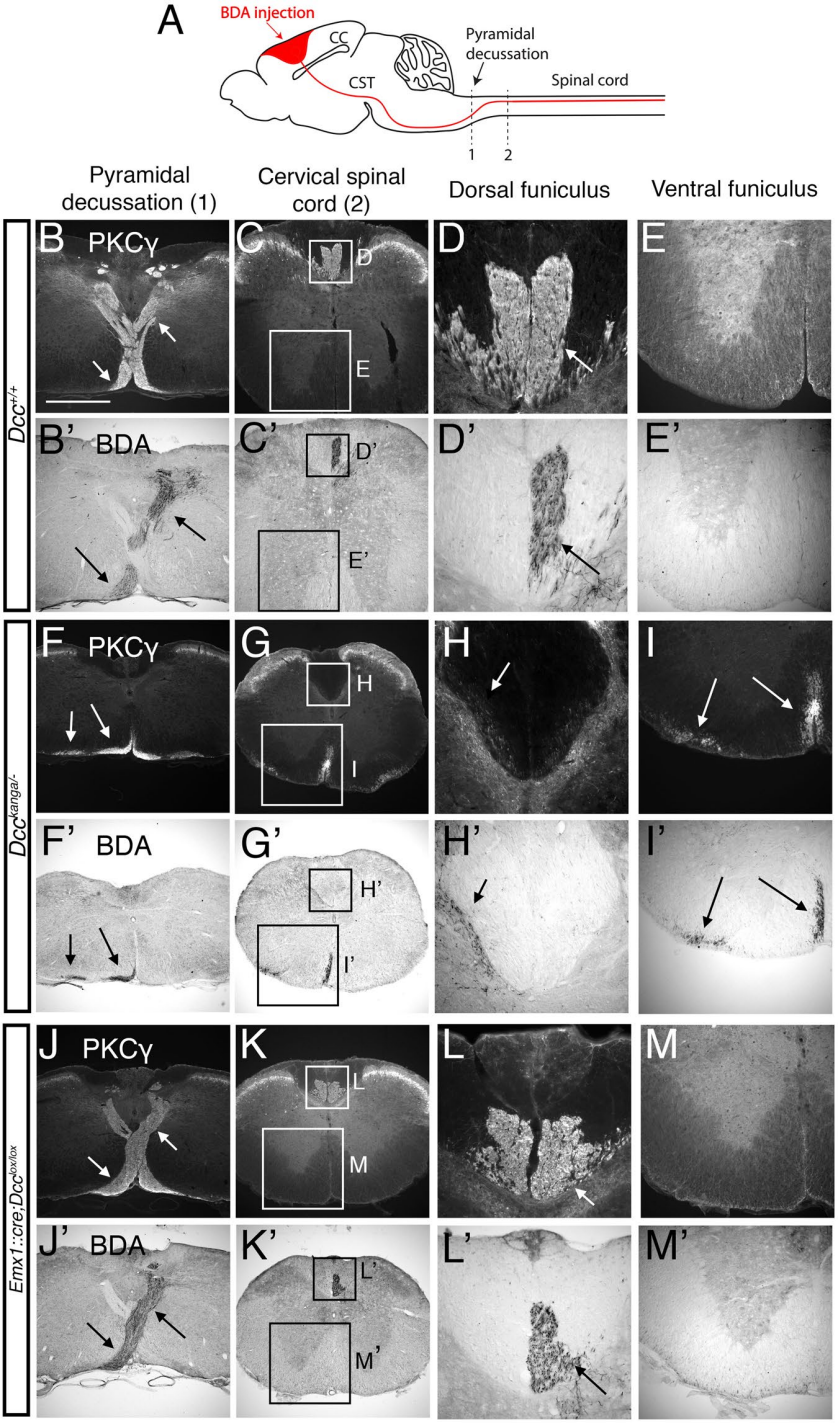
Abrogation of neocortical DCC expression fails to reproduce the abnormal pyramidal decussation observed in *Dcc* ^*kanga*/*−*^ mice. (**A**) Schematic representation of a sagittal section through an adult mouse CNS indicating the trajectory of the CST and the level of the coronal sections presented in this figure. Biotin dextran amine (BDA) was injected into the left motor cortex of *Dcc* ^+/+^ (n = 4; **B**–**E**, B’–E’), *Dcc* ^*kanga*/*−*^ (n = 4; **F–I**; F’–I’) and *Emx1*::*cre*;*Dcc* ^*lox*/*lox*^ (n = 3; **J–M**, J’–M’) mice to label the left-sided CST axons. The CST was visualized on coronal sections at the level of the pyramidal decussation and in the spinal cord, by immunostaining against the PKCγ (visualization of the two CSTs; B–M), or by revelation of the BDA tracer (visualization of the left-hand CST alone; B’–M’). The CST trajectory was similar in *Dcc* ^+/+^ (**B–E**) and *Emx1*::*cre*;*Dcc* ^*lox*/*lox*^ (**J–M**) mice. In *Dcc* ^*kanga*/*−*^ mice, the CST axons did not cross the midline at the pyramidal decussation, but spread in two bundles, one lateral and the other medial, that remained in the ventral ipsilateral spinal cord (**F–I**). The scale bar represents 336 μm in B, C, F, G, J, K; 168 μm in E, I, M; and 84 μm in D, H, L.

After validating the tools, we compared the CST anatomy of *Emx1*::*cre*;*Dcc* ^*lox*/*lox*^ *mice* with various *Dcc* deficient mouse lines and their respective controls. *Dcc* ^*kanga*/*−*^ mice were compared to *Dcc* ^*kanga*/+^ mice. *Dcc* ^+/−^ mice with DCC haploinsufficiency were compared to *Dcc* ^+/+^ mice. The CST organization was investigated by PKCγ immunostaining and by unilateral BDA injection into the left motor cortex (Fig. 7). In *Dcc* ^+/+^ control mice (n = 4/4), BDA-labeled CST axons crossed the midline at the pyramidal decussation (Fig. 7B,B’), then turned dorsally and continued their trajectory into the dorsal funiculus of the contralateral spinal cord (Fig. 7B–E,B’–E’). In *Dcc* ^+/−^ mice (n = 5/5, Supplementary Figure 2A–D’) and in *Dcc* ^*kanga*/+^ mice (n = 4/4, Supplementary Figure 2E–H’), the anatomy of the CST at the pyramidal decussation was similar to that of Dcc^+/+^ mice. As expected, *Dcc* ^*kanga*/*−*^ mice (n = 4/4) had major anomalies of the CST. Indeed, at the pyramidal decussation, CST axons completely failed to cross the midline, and three distinct fasciculi were observed within the ipsilateral spinal cord (Fig. 6F,F’,G,G’): (i) a minor group of axons located in the ventral part of the dorsal funiculus (Fig. 7G,G’,H,H’); (ii) a bundle in a ventro-medial position (Fig. 7I,I’); and (iii) a bundle in a ventro-lateral position (Fig. 7I,I’). These findings were reminiscent of what had been observed in *Dcc* ^*kanga*/*kanga*^ mice^8^, emphasizing the importance of DCC in CST guidance at the midline. To further characterize the CST of *Dcc* ^*kanga*/*−*^ mice, we combined unilateral CST labeling with 3DISCO optical clearing and light-sheet microscopy of adult brains and spinal cords. In *Dcc* ^*kanga*/+^ mice (n = 1/1, Supplementary Movie 6), as well as in *Emx1*::*cre*;*Dcc* ^*lox*/*lox*^ (n = 3/3, Supplementary Movie 7) the CST axons crossed the midline and turned dorsally at the decussation. No axons crossing the midline were detected in *Dcc* ^*kanga*/*−*^ mice (n = 1/1); the CST axons instead formed two bundles, one lateral and the other medial, that remained in the ventral ipsilateral spinal cord (Supplementary Movie 8). These results further support the role of DCC in CST axon guidance at the level of the pyramidal decussation.

Interestingly, the pathway followed by CST axons was normal in *Emx1*::*cre*;*Dcc* ^*lox*/*lox*^ mice (n = 3/3), in which CST axons crossed the midline at the level of pyramidal decussation and continued their trajectory through the dorsal funiculus of the contralateral spinal cord (Fig. 6J–M,J’–M’). These results demonstrate that DCC deletion in the neocortex (and thus in the CST) is not sufficient to induce abnormal pyramidal decussation. The role of DCC in CST axon guidance at the midline is therefore non cell-autonomous.

## Discussion

We demonstrate that DCC deficiency is associated with abnormal CST midline crossing and a reduced ability to generate asymmetric movements in both humans and mice. In addition, we show that selective suppression of DCC expression in the mouse neocortex does not affect the pyramidal decussation, demonstrating that the role of DCC in CST axon guidance at the midline is non cell-autonomous in mice.

Using a combination of TMS and DTI tractography, we obtained evidence that *DCC*-CMM patients have abnormal CST midline crossing at the level of the pyramidal decussation. Our TMS findings are consistent with those of previous TMS studies of two *DCC*-CMM patients^20–22^. In all six of the *DCC*-CMM patients studied here, stimulation of the motor cortex hand representation elicited ipsilateral MEPs, whereas in healthy volunteers the MEPs are strictly contralateral to the stimulated hemisphere^18, 35–37^. MEPs elicited by stimulation of the motor cortex with TMS result from the transmission of a neuronal signal from the cortex to the peripheral muscles via fast-conducting CST fibers and spinal motoneurons^38–40^. Direct connections of CST axons to motoneurons (direct cortico-motoneuronal connections) make a significant contribution to MEPs in humans^41–44^. In our patients, the presence of ipsilateral MEPs with latencies similar to those of contralateral MEPs indicates direct connections of CST axons from one hemisphere to hand muscle motoneurons on both sides of the spinal cord. These results are corroborated by DTI analysis of two of the *DCC*-CMM patients, in whom we found more uncrossed CST fibers than crossed CST fibers, contrary to the situation observed in control subjects. Our multimodal study strongly suggests that abnormal CST midline crossing, rather than aberrant CST branching in the spinal cord, is responsible for the abnormal ipsilateral MEPs observed in *DCC*-CMM patients.

The phenotype severity varies among the six DCC patients, but this phenotypic variation was not linked to the genotype. For example, patients 1 and 6 belong to the same family and have different phenotypes despite carrying the same mutation (Table 1).

In five of the six *DCC* patients, stimulation of the dominant hemisphere evoked ipsilateral responses in 100% of the pulses. In four of these patients, the amplitude of the ipsilateral MEPs was higher than that of the contralateral MEPs. In a previous study of seven *RAD51*-CMM patients, we found that the frequency of ipsilateral MEPs was always below 100% and that their amplitude was always smaller than that of normal contralateral MEPs^17^. In patients with X-linked Kallmann syndrome with mirror movements, the relative amplitude of the ipsilateral and contralateral MEPs was variable across individuals^45^. The relative proportion of ipsilateral and contralateral CST projections is likely to be the main determinant of the relative amplitudes of contralateral and ipsilateral MEPs^40, 44, 45^. In keeping with this hypothesis, *DCC*-CMM patients would have a majority of ipsilateral CST projections.

We found an absence of pyramidal decussation in *Dcc* ^*kanga*/*−*^ mice, as previously described in *Dcc* ^*kanga*/*kanga*^ mice^8^. The pyramidal decussation is partial in human *DCC*-CMM patients, normal in *Dcc* ^+/−^ mice and completely absent in *Dcc* ^*kanga*/*−*^ mice. While 95% of CST axons cross the midline in rodents^46–48^, this proportion varies between 75% and 90% in humans with important inter-individual differences^1, 49, 50^. Despite species-related differences regarding the anatomy of the pyramidal decussation, our data suggest a role for DCC in CST guidance at the midline in both mice and humans.

Besides the description of the hopping gait and ataxia^8^, careful investigation of the motor behavior of *Dcc* ^*kanga*/*−*^ mice had not been performed. Here, we show that *Dcc* ^*kanga*/*−*^ mice exhibit very specific motor impairments, as they generate symmetric movements during stereotypic locomotion (hopping gait) or voluntary symmetric forelimb movements (exploratory reaching behaviors). In *Dcc* ^*kanga*/*−*^ mice, DCC deficiency not only impacts on CST and CC development but also affects other DCC-expressing cell populations, such as commissural spinal cord interneurons, that are critical for locomotion^12^. Two recent studies used conditional knockout EphA4 mice to dissect the neuronal circuits responsible for their hopping gait. In *Emx1*::*cre*;*EphA4* ^*flox*/*flox*^ mice, conditional EphA4 deletion in the forebrain resulted in normal stereotypic locomotion despite bilateral CST projections to the spinal cord^23, 24^. In contrast, specific EphA4 deletion in the spinal cord or in glutamatergic interneurons was sufficient to induce hopping locomotor activity^23^. Together, these results show that stereotypic left-right alternation relies on spinal commissural circuits rather than on proper CST wiring. The exploratory reaching test provides a better behavioral paradigm to properly evaluate the role of the CST in motor lateralization. *Emx1*::*cre*;*EphA4* ^*flox*/*flox*^ mice, which have bilateral CST projections to the spinal cord as human CMM patients, exhibit symmetric voluntary movements during this test^23–25^. This abnormal symmetrical reaching behavior might thus represent a good equivalent of human MM. *Dcc* ^*kanga*/*−*^ mice have a similar motor phenotype despite their complete lack of pyramidal decussation. Mirror movements have not been described in patients with horizontal gaze palsy with progressive scoliosis (*ROBO3* mutations), who lack the pyramidal decussation and have a completely uncrossed CST, demonstrating that mirror movements are related to bilateral spinal cord projections arising from a single primary motor cortex^18, 51^. By contrast, MM have been reported in one patient with Klippel-Feil syndrome and one patient with CMM both completely lacking the pyramidal decussation, suggesting that the uncrossed CST axons might have an aberrant bilateral branching within the spinal cord^52, 53^. Two hypotheses might thus be proposed to explain the discrepancy between the lack of pyramidal decussation and the abnormal symmetrical reaching behavior in *Dcc* ^*kanga*/*−*^ mice: (i) bilateral CST projection to the spinal cord might be sufficient but not necessary to induce symmetrical reaching behaviors, or (ii) uncrossed CST axons might branch bilaterally within the spinal cord.

In mice lacking a functional DCC, CST axons fail to cross the midline at the level of the pyramidal decussation. Selective suppression of DCC in the mouse neocortex, and thus in the CST, did not alter CST midline crossing revealing a non cell autonomous effect of DCC on CST. One potential limitation of these findings stems from unresolved issues on the exact nature of the DCC kanga allele. Indeed, the *Dcc* ^*kanga*/*kanga*^ seemed to be lethal in our hands whereas the *Dcc* ^*kanga*/*−*^ that we used in this study was viable. This might indicate that the DCC kanga allele is more severe than the null allele through a dominant negative effect. However, as described previously^8, 13^, we did not detect DCC in brainstem CST axons during normal development (whereas we detected it on fasciculus retroflexus), suggesting DCC influences CST midline crossing indirectly. Altogether, these results demonstrate that the role of DCC in CST axon guidance at the midline is non cell-autonomous. Very few axon guidance receptors have been reported to have non cell-autonomous functions. The WNT binding receptor Frizzled3 has a non cell-autonomous role in guiding medium spiny neurons in mice, possibly by positioning corridor guidepost cells^54^. In *Drosophila*, the DCC ortholog Frazzled1 is required for the guidance of retinal cells and longitudinal axons in a non cell-autonomous manner, possibly by controlling Netrin-1 distribution and presentation^55, 56^.

Many CNS commissural neurons that express DCC fail to cross the midline in *Dcc* mutants^6–9, 12, 13^, suggesting a cell-autonomous role for this receptor in this setting. Our work shows for the first time that DCC controls CST midline crossing in a non cell-autonomous manner and unravels a new level of complexity in the role of DCC in axon guidance at the midline.

## Material and Methods

### Subjects

Six right-handed CMM patients (3 males and 3 females) with documented mutations in the *DCC* gene^14, 16^ (see Table 1) were matched for age, gender and handedness with six healthy volunteers. The severity of mirror movements was evaluated with the Woods and Teuber rating scale^57^. All the participants gave their written informed consent and the protocol was approved by the CPP Ile-de-France 6 (2013-A00616-39). All the experiments were performed according to this protocol. Patients gave their written informed consent for videos appearing in the publication.

### Electrophysiological experiments

EMG signals were recorded bilaterally from the first dorsal interosseous (FDI) muscles^17^. Motor evoked potentials (MEPs) induced by single monophasic pulses delivered with a figure-of-eight coil connected to a Magstim 200 (Magstim, Dyfed, UK) were recorded from electromyographic signals. Coil positioning for the stimulation of the FDI muscles in M1 and measurements of the resting motor threshold were previously described^17^. Between 30 and 60 MEPs evoked by calibrated stimulation (1.3x the resting motor threshold) of the dominant hemisphere were recorded bilaterally in the FDI muscles to compare the frequency, latency and amplitude of the normal contralateral MEPs with those of any mirror MEPs recorded in the hand ipsilateral to the stimulation site.

### Magnetic resonance imaging (MRI)

MRI was performed with a Siemens 3 T MAGNETOM Verio with a 32-channel head coil. The MRI protocol included anatomical three-dimensional (3D) T1-weighted MPRAGE images (TR = 2.3 s; TE = 4.18 ms; flip angle = 9°; TI = 900 ms; voxel size = 1 × 1 × 1 mm^3^; 176 slices) and spin-echo echo-planar diffusion tensor imaging (TR = 10 s, TE = 87 ms, voxel size = 2 × 2 × 2 mm^3^, 60 slices, 60 gradient encoded directions with a b value of 1500 s/mm^2^, 11 non diffusion-weighted volumes).

### Tractography analysis

Tractography analysis was performed in two patients (#1 and 6).

Diffusion images were preprocessed as previously described with MRtrix software^17^. Raw diffusion-weighted data were corrected for motion and geometric distortions secondary to eddy currents by using a registration technique based on the geometric model of distortions^58^. The fiber orientation distribution function (ODF) was estimated by using the constrained spherical deconvolution (CSD) method in MRtrix. The sufficient angular resolution allowed high-order fiber orientation estimation algorithms. The ODF information obtained from CSD was used, with a suitable fiber-tracking algorithm, to infer the connectivity of crossing fibers. We used a probabilistic streamlines algorithm with the entire ODF as a probability density function (ODF threshold = 0.1; step size = 0.2 mm as 1/10 of the voxel size; radius of curvature = 1 mm; up-sampling of DWI data to 1 mm). In the native individual space, we performed seed-to-target analysis in regions of interest defined along the CST^17^. These regions included the anterior bundle of the CST in the upper part of the brainstem, the lower part of the brainstem, and the lateral horn of the spinal cord (see Fig. 2A). We used a probabilistic tractography algorithm: the number of fibers connecting a seed voxel to a target voxel was calculated by sampling one million draws for each fiber connecting the seed to the target. The CST tracts (the normally crossed CST and the abnormally uncrossed CST) were reconstructed for each subject. We analyzed the proportion of crossed versus uncrossed portions of the CST by using a laterality coefficient (NF Crossed − NF Uncrossed)/(NF Crossed + NF Uncrossed), where NF is the number of fibers. Ratios closer to 1 indicate greater crossed than uncrossed CST, whereas ratios closer to −1 indicate greater uncrossed than crossed CST.

### Animals and genotyping

*Dcc* ^7^, *Dcc* ^*kanga*^ ^8^ and *Dcc* ^*lox*^ ^26^ mice, as well as the *Emx1*::*cre* ^27^ and *Tau* ^*GF*^ ^31^ lines have previously been described and genotyped by PCR. Except for the *Dcc* ^*kanga*^ line, all the mouse lines were maintained on a C57B/6 J background. *Dcc* ^*kanga*^ mice arose in a C.AKR-Tgn^cog^ research mouse colony at The Jackson Laboratory, and the Tgn^cog^ mutation has been segregated out of the line^8^. The day of the vaginal plug was E0, and the day of birth was P0. All animal procedures were approved by the Regional Animal Experimentation Ethics Committee C2EA-05 Charles Darwin and the French Ministère de l’éducation nationale de l’enseignement supérieur et de la recherche (project N°01558.03). We strictly performed these approved procedures.

### Behavioral study of Dcc deficient mice

All the behavioral studies were performed blindly to the genotype. The tests were performed on male and female mice aged between 8 and 12 weeks. For the first three days, the mice were habituated to being handled by the experimenters in order to limit stress. Mice were then tested with a partial SHIRPA protocol (grasping, clasping and auditory tests were performed, and whisker state was evaluated) in order to rule out major neurological abnormalities.

*The open field test* was used to evaluate spontaneous activity and locomotion: mice were placed in the center of a 0.25-m^2^ arena and allowed to explore freely for 5 minutes. During this time, they were tracked and recorded with a camera fixed above the arena, and the total walking distance was calculated with Topscan software.

*The Ladder test* apparatus (Locotronic) consists of a 124 cm × 8 cm corridor with a floor composed of 78 bars each 1 cm apart. The mice were made to cross the corridor, and the number of slips of the forelimbs, hindlimbs and tail was automatically detected by 158 infrared captors placed on the corridor walls (sampling frequency 1000 Hz). This test evaluates the precision and coordination of limb positioning.

#### Treadmill

Mice were placed on a transparent treadmill (14 cm × 6 cm) moving at 12 cm/s. After a short training session, the mice had to run for ten seconds, during which period the positioning of their paws was recorded by a camera fixed under the apparatus. The numbers of symmetric and asymmetric strides were counted after excluding frames in which the mouse was not running.

#### Rotarod

The accelerating Rotarod (BIOSEB) consists of a horizontal rod 3 cm in diameter, turning on its longitudinal axis. The training phase consisted of walking on the rod at a rotational speed varying from 4 to 40 rpm for one minute. The mice were then subjected to four trials in which the speed of rotation increased gradually from 4 rpm to 40 rpm over 5 min. Time spent on the rod was recorded and averaged for the 4 trials. The test was repeated three days in a row with the same procedure, except that the training session was performed only on the first day.

#### Grip test

Forepaw muscle strength was measured with a grip test. The mouse was held by the base of its tail and allowed to firmly grab the grid of the device with its forepaws. The mouse was then pulled gently backwards until it released its grip. The peak force (N) in each trial was considered as the grip strength. Four successive measurements were averaged. The same procedure was performed with forepaws and hindpaws at the same time.

#### Reaching exploratory behavior

When placed in a new environment, as a glass cylinder, mice engage in “reaching” exploratory behavior, in which they contact walls with their forepaws^24^. This contact can be made with the two paws simultaneously (symmetric movement) or independently (asymmetric). Ten reaching movements were recorded with a video camera and then examined frame-by-frame to calculate the numbers of asymmetric and symmetric movements.

### Statistical analysis

Data were analyzed with SPSS statistical software version 22.0 (Chicago, Illinois, USA). The normality of variable distributions and the homogeneity of variance across the groups were assessed with the Kolmogorov-Smirnov and Levene tests, respectively. Variables that failed any of these tests were analyzed with the nonparametric Mann–Whitney test. Variables that passed the normality test were analyzed with ANOVA followed by the Bonferroni *post hoc* test for multiple comparisons, or with Student’s *t* test when comparing two groups. Paired data were analyzed by repeated-measures ANOVA with two factors, followed by the Bonferroni *post hoc* test for multiple comparisons. Categorical variables were compared using Pearson’s χ^2^ test or Fisher’s exact test.

### Immunohistochemistry

All the immunohistochemistry experiments were performed on at least 3 animals per age and per genotype. For light microscopy, P0-P2 mice were anesthetized on ice and adult mice were anesthetized with sodium pentobarbital (50 mg/kg i.p.). Embryos were fixed by immersion in 4% paraformaldehyde with 0.12 M phosphate buffer pH 7.4, and post-natal mice were perfused through the aorta with 0.12 M phosphate buffer, pH 7.4, containing 4% paraformaldehyde. Tissue preparation and immunostaining were carried out as described in ref. 59, using the following primary antibodies: goat anti-DCC (1/100; Santa Cruz Biotechnology, Santa Cruz, California); rabbit anti-PKCγ (1/100, Santa Cruz) to reveal the CST in mature mice after P2; chicken anti-GFP (1/500, Aveslab) to reveal the CST in both newborn and mature *Emx1*::*cre*;*Tau* ^*mGFP*^ mice ; mouse anti-MBP (1/200, Chemicon, Millipore, Molsheim, France) to reveal corpus callosum in adult mice and rat anti-L1 (1/400, Millipore) to reveal corpus callosum before myelination.

### Tracing of the corticospinal tract

#### Surgery and sample collection

Adult male or female mice were anesthetized with a mixture of ketamine (146 mg/kg) and xylazine (7.4 mg/kg) and placed in a stereotaxic frame. Pressure injections of an anterograde tracer (biotinylated dextran amine, BDA, MW 10 000, SIGMA) targeting the left motor cortex were performed. Six 0.2-μl aliquots of 10% BDA solution in normal saline were injected (0.1 μl/min) with a 10-µl Hamilton microsyringes fitted with a removable needle (Hamilton, 7762-03) at the following coordinates, as determined in ref. 60: (i) A (anteriority: positive values are rostral to bregma, negative values are caudal to bregma) = 1, L (laterality to bregma) = 2, D (depth from the surface of the skull) = 1; (ii) A = 1, L = 1, D = 1; (iii) A = −0.25, L = 2, D = 1; (iv) A = −0.25, L = 1, D = 1; (v) A = −1, L = 2, D = 1; and (vi) A = −1, L = 1, D = 1. At each injection point, the needle was left in place for 3 min before and after the injection to minimize leakage. After surgery, the wound was cleansed and the skin sutured. Fourteen days following BDA injections, the mice were deeply anesthetized and perfused as described for immunohistochemistry procedure.

#### Revelation of BDA labeling after cryostat sectioning

The brain and spinal cord were treated as described above for the immunohistochemistry procedure^59^. Coronal sections 30 μm thick through the entire brain and spinal cord were cut on a cryostat. Sections were washed for 15 min in 0.1 M PBS pH 7.3 and incubated for one hour in PBSGT (PBS containing 0.25% Triton-X, 0.2% gelatin) and lysine (0.1 M). The sections were then incubated overnight in streptavidin-complex conjugated to horseradish peroxidase solution (HRP, 1/400, Sigma) in PBSGT. The sections were washed 4 × 10 min in PBST, then incubated for 40 min in 1% DAB solution (3,3′-diaminobenzidine, Sigma-Aldrich) in 0.1 M Tris pH 7.6 containing 0.015% H_2_O_2_. The sections were washed 3 × 5 min in 0.1 M Tris in order to stop the reaction, then dehydrated before mounting in Eukitt (Sigma). Images were acquired with a DMR Leica microscope.

#### 3DISCO clearing and light-sheet imaging

We used the 3DISCO clearing, 3D light-sheet imaging and image processing procedures as previously described^10^.

## Electronic supplementary material

Supplementary information

Supplementary Movie 1

Supplementary Movie 2

Supplementary Movie 3

Supplementary Movie 4

Supplementary Movie 5

Supplementary Movie 6

Supplementary Movie 7

Supplementary Movie 8
