# Supplementary material for: Non cell-autonomous role of DCC in the guidance of the corticospinal tract at the midline
Source: Sci Rep. 2017 Mar 24;7:410. doi: 10.1038/s41598-017-00514-z (PMC5428661; doi:10.1038/s41598-017-00514-z)
Supplement: Supplementary file 1 — Supplementary information [file 41598_2017_514_MOESM1_ESM.pdf]

Non cell-autonomous role of DCC in the guidance of the corticospinal tract at the midline

Quentin Welniarz,[1,2] Marie-Pierre Morel,[2] Oriane Pourchet,[1,2] Cécile Gallea,[1] Jean-Charles Lamy,[1] Massimo Cincotta,[3] Mohamed Doulazmi,[4] Morgane Belle,[5] Aurélie Méneret,[1,7] Oriane Trouillard,[1] Marta Ruiz,[1] Vanessa Brochard,[6] Sabine Meunier,[1] Alain Trembleau,[2] Marie Vidailhet,[1,7] Alain Chédotal,[5] Isabelle Dusart,[2*] and Emmanuel Roze[1,7*]

[1] Sorbonne Universités, UPMC Univ Paris 06, INSERM U 1127, CNRS UMR 7225, Institut du Cerveau et de la Moelle épinière, F-75013, Paris, France

[2] Sorbonne Universités, UPMC Univ Paris 06, INSERM, CNRS, Institut de Biologie Paris Seine, Neuroscience Paris Seine, F-75005, Paris, France

[3] Unità Operativa di Neurologia-Firenze, Azienda USL Toscana Centro, Ospedale San Giovanni di Dio, 50143 Firenze, Italy

[4] Sorbonne Universités, UPMC Univ Paris 06, INSERM, CNRS, Institut de Biologie Paris Seine, Adaptation Biologique et vieillissement, F-75005, Paris, France

[5] Sorbonne Universités, UPMC Univ Paris 06, INSERM, CNRS, Institut de la Vision, F-75012, Paris, France

[6] Centre d'Investigation Clinique 14-22, INSERM/AP-HP, Paris, France

[7] Département de Neurologie, AP-HP, Hôpital Pitié Salpêtrière, Paris, France

[*] *Equal contributions*

Corresponding author: Emmanuel Roze

Département des maladies du système nerveux, hôpital Pitié Salpêtrière,

47-83 boulevard de l'Hôpital, 75013 Paris

E-mail: emmanuel.flamand-roze@psl.aphp.fr

Phone: +33142162748; Fax: +33142162474

Supplementary information

Supplementary Movie 1. A 41-year-old woman with typical congenital mirror movements associated with *DCC* mutation was asked to make finger movements with only one hand.

Supplementary Movie 2. Movie of an adult $Dcc^{kanga/-}$ mouse on a treadmill. Note the symmetric movements of both the forelimbs and hindlimbs during locomotion.

Supplementary Movie 3. Movie of an adult $Dcc^{kanga/+}$ mouse on a treadmill. Note the asymmetric movements of both the forelimbs and hindlimbs during locomotion.

Supplementary Movie 4. Movie of an adult $Dcc^{kanga/+}$ mouse establishing asymmetric forelimb contacts with the wall during the exploratory reaching test.

Supplementary Movie 5. Movie of an adult $Dcc^{kanga/-}$ mouse establishing symmetric forelimb contacts with the wall during the exploratory reaching test.

Supplementary Movie 6. 3D movie of the CST at the level of the pyramidal decussation in an adult $Dcc^{kanga/+}$ mouse. The CST was labeled with BDA and revealed with streptavidin-CY5. Note how all the CST axons cross the midline and turn dorsally.

Supplementary Movie 7. 3D movie of the CST at the level of the pyramidal decussation in an adult *Emx1::Cre;*$Dcc^{lox/lox}$ mouse. The CST was labeled with BDA and revealed with streptavidin-CY5. Note how all the CST axons cross the midline and turn dorsally.

Supplementary Movie 8. 3D movie of the CST at the level of the pyramidal decussation in an adult $Dcc^{kanga/-}$ mouse. The CST was labeled with BDA and revealed with streptavidin-CY5. Note how the CST axons fail to cross the midline and remain in the ipsilateral spinal cord, forming two ventral bundles.

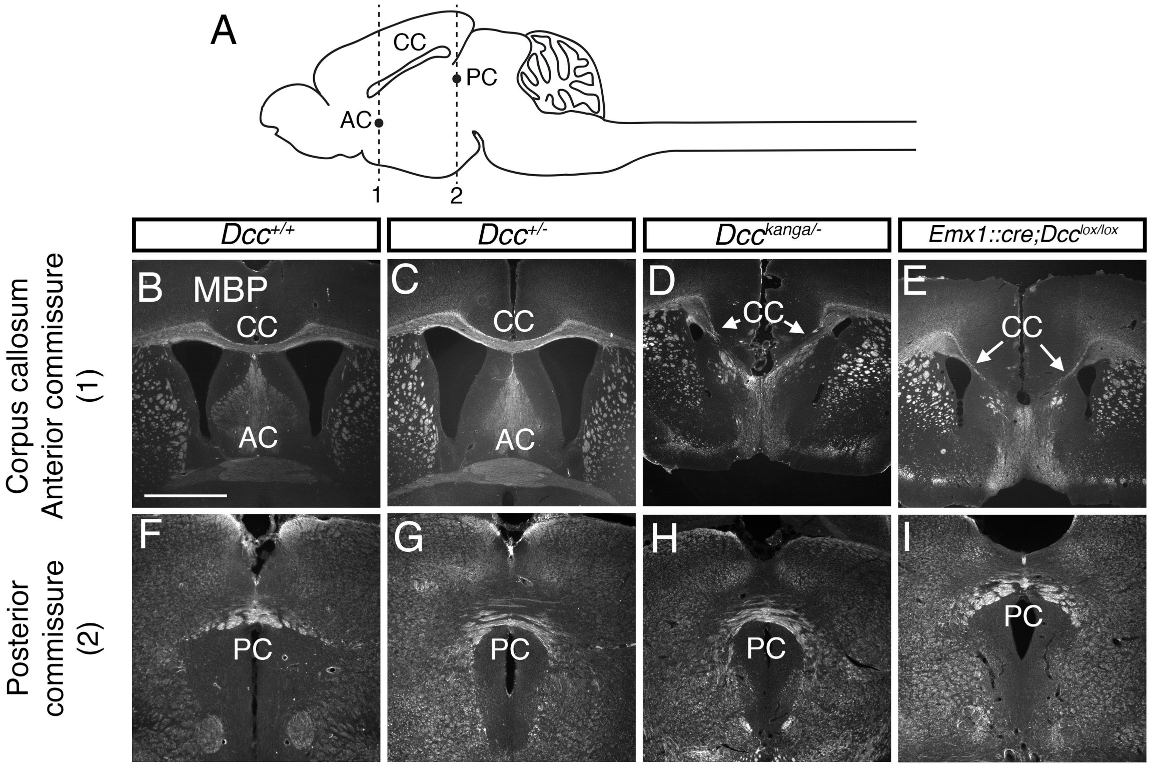

Supplementary Figure 1. Abrogation of neocortical DCC expression reproduces the lack of forebrain commissures observed in *Dcc*$^{kanga/-}$ mice. (A) Schematic representation of an adult mouse sagittal section indicating the level of the coronal sections presented in this figure. The corpus callosum (CC; B-E), anterior commissure (AC; B-E), and posterior commissure (PC; F-I) were revealed by myelin basic protein (MBP) immunostaining. The CC and AC were present in *Dcc*$^{+/-}$ mice (n = 4), as in *Dcc*$^{+/+}$ mice (n = 4; A-B). Note the absence of CC and AC in both *Dcc*$^{kanga/-}$ (n = 3; D) and *Emx1::cre;Dcc*$^{lox/lox}$ mice (n = 3; E). The posterior commissure was present in all *Dcc* mutants (F-I). The scale bar represents 672 µm in B-E and 336 µm in F-I.

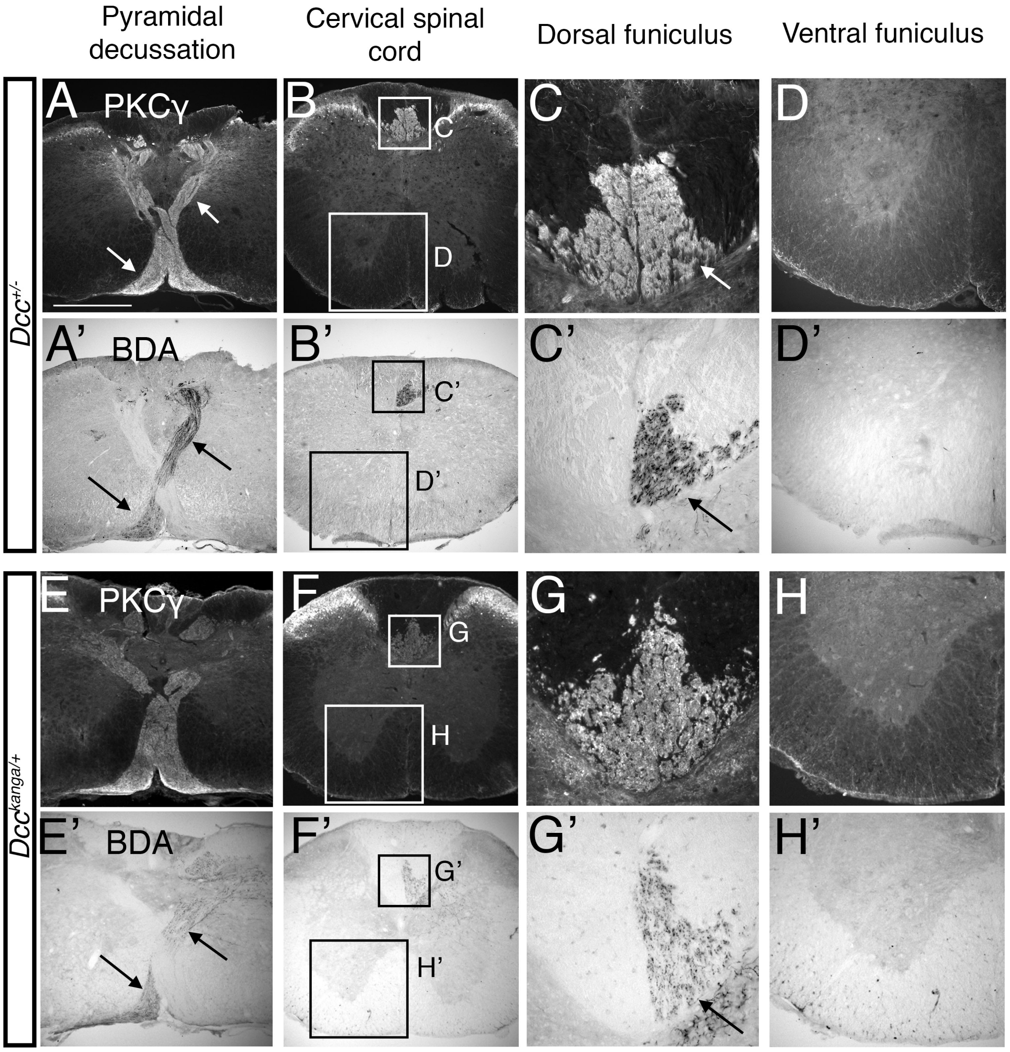

Supplementary Figure 2. The anatomy of the pyramidal decussation is normal in *Dcc*$^{+/-}$ and *Dcc*$^{kanga/+}$ mice. BDA was injected into the left motor cortex of adult *Dcc*$^{+/-}$ mice (n = 5, A-D') and *Dcc*$^{kanga/+}$ mice (n=4, E-G') to label the left CST axons. The CST was visualized on coronal sections at the level of the pyramidal decussation and in the spinal cord by PKCγ immunostaining (visualization of the two CSTs; A-H), or by revelation of the BDA tracer (visualization of the left CST alone; A'-H'). The CST trajectory is normal in *Dcc*$^{+/-}$ mice and *Dcc*$^{kanga/+}$ mice: the CST axons cross the midline at the decussation (A, A', E, E'), turn dorsally and continue their trajectory in the dorsal funiculus of the contralateral spinal cord (B-D, B'-D', E-H, E'-H'). The scale bar represents 336 µm in A, A', B, B', E, E', F, F'; and 168 µm in D, D', H, H'; 84 µm in C, C', G, G'.
